# Visible-light-induced copper-mediated reversible deactivation radical polymerisation without additional photocatalysts

**DOI:** 10.1039/d5sc05171a

**Published:** 2025-08-28

**Authors:** Mia D. Hall, Boyu Zhao, Evelina Liarou, Tanja Junkers, David Haddleton

**Affiliations:** a University of Warwick, Department of Chemistry Library Road Coventry CV4 7AL UK d.m.haddleton@warwick.ac.uk; b Polymer Reaction Design Group, School of Chemistry, Monash University 19 Rainforest Walk Clayton VIC 3800 Australia tanja.junkers@monash.edu

## Abstract

Photochemistry mediated by visible light is attractive as it avoids the limitations of higher energy UV light. Photo-induced copper-mediated reversible deactivation radical polymerisation (photo Cu-RDRP) typically requires both a copper catalyst and a separate photocatalyst (PC) to exploit lower energy irradiation. However, by covalently anchoring a PC to another reagent, dual functionality can be established. Herein, a simiplified approach is demonstrated using a modified commerically available dye as an initiator and PC simultaneously. A visibly fluorescent and highly absorbing dye, Hostasol Yellow, is chemically incorporated into a polymer following irradiation under six different UV and visible light wavelengths, resulting in high monomer conversion (>90%) and polymers with low dispersity (≤1.13). The wavelength-dependent behaviour of the dye was probed, reaffirming a mismatch between reactivity and absorptivity. Kinetic studies show high monomer conversions within just a few minutes, with good end-group fidelity confirmed by *in situ* chain extension and matrix-assisted laser desorption/ionization time-of-flight (MALDI-ToF). The versatility of this PC initiator was extended to give a wide variety of molecular weights (2700–420 000 g mol^−1^) and different hydrophobic, hydrophilic and semi-fluorinated polyacrylates.

## Abstract 2

Photochemistry mediated by visible light is attractive as it avoids the limitations of higher energy UV light.

## Graphical abstract

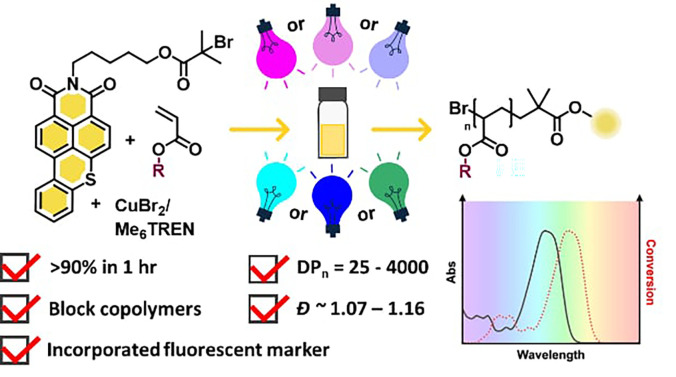

## Introduction

Controlled reversible deactivation radical polymerisation (RDRP) techniques such as atom transfer radical polymerisation (ATRP),^1–3^ single electron transfer living radical polymerisation (SET-LRP),^4,5^ reversible addition fragmentation chain-transfer polymerisation (RAFT),^6^ and nitroxide-mediated polymerisation (NMP),^7^ have revolutionised polymer chemistry by facilitating the synthesis of well-defined polymers with tolerance to functional groups and conditions often not achievable by traditional living polymerisation.^8–13^ Considerable research has focused on externally regulating these polymerisations, such as through the use of light,^14–18^ electrical currents,^19^ and mechanical force.^20^ Photochemically triggering a RDRP allows for arguably more environmentally-friendly conditions, as well as spatiotemporal control and unlimited accessibility to the stimulus, namely light.^21,22^

Conventional photo-induced RDRP techniques often require the use of high energy UV irradiation (*e.g.*, *λ*_max_ ∼ 365 nm), which exhibits limited light penetration and can lead to the formation of side products.^23^ While UV has been previously acknowledged as a highly suitable region for photo Cu-RDRP due to copper complex absorption, recent work has suggested conversion does not align with the catalyst absorption spectrum, resulting in red-shift action plots.^24–26^ This has increased interest in the use of lower energy irradiation for polymerisations where the absorption is relatively low compared to absorption maxima.^27,28^

The use of higher energy visible light (<500 nm) has been well documented for RAFT, particularly for photoinitiated and photoiniferter systems.^29–33^ However, photoinduced electron/energy transfer (PET) RAFT has shown the ability to use lower energy wavelengths up to the near infra-red (NIR), with applications ranging from 3D printing to synthesis through skin barriers.^34–39^ These exemplary studies on PET-RAFT utilise organo-dyes, which are often visibly fluorescent, with strong absorbance in the visible region and long-lived excited states.^37,40^ Metal-free ATRP has also employed these dyes to facilitate polymerisation under lower energy irradiation.^41,42^ Recently, fluorescent organic dyes and metal-containing porphyrins have been implemented in photo Cu-RDRP allowing for polymerisation under lower energy wavelengths whilst maintaining good polymerisation control.^43–48^

Numerous papers document the integration of fluorescent dyes into different RDRP components, namely monomers,^49–52^ ATRP initiators,^28,52–56^ and RAFT agents,^40^ leading to well-defined labelled polymers. These polymers are often used for observation and analysis *via* fluorescence microscopy and laser scanning confocal microscopy.^57^ This is particularly useful for studying polymers in biological systems, with tracking *in vivo* with favourable spatiotemporal resolution.^57–59^

Interestingly, no literature until very recently has attempted to exploit a fluorescently tagged RDRP component as a dual low energy photocatalyst (PC). Zhang and colleagues found that by conjugating a fluorophore, either fluorescein or boron-dipyrromethene (BODIPY), to a RAFT agent, a controlled polymerisation occurred under lower energy wavelengths.^40^ However, only wavelengths up to 470 nm were tested and while fluorescein displayed photo-instability, BODIPY required multiple synthesis steps with low yields. Judzewitsch and colleagues prepared a zinc tetraphenyl porphyrin monomer and embedded it into a polymer, referred to as a self-catalyst for PET-RAFT and photosensitiser for anti-microbial applications,^60^ while Zhu and others designed a similar monomer which was polymerised alongside glycomonomers.^61^

PCs have also been modified into photocatalytic heteroinitiators to facilitate photo-induced Cu-RDRP under low-energy visible light. Diacon and others synthesised a perylenediimide-based two-arm initiator to polymerise methacrylates under white light for thermoresponsive grafting, which resulted in relatively low monomer conversion due to slow initiation.^62^ Lin and colleagues amended zinc porphyrin into a four-arm initiator to polymerise glycomonomers under yellow light irradiation, which formed well-defined polymers but with only moderate agreement between the theoretical and experimental molecular weights (MWt).^63^ Therefore, despite the extensive use of fluorescent-labelled initiators and monomers in both thermal and photo Cu-RDRP, no work yet exploits the fluorescent nature of a simple dye derivative with a single initiating unit as a dual RDRP PC.

**Fig. 1:**
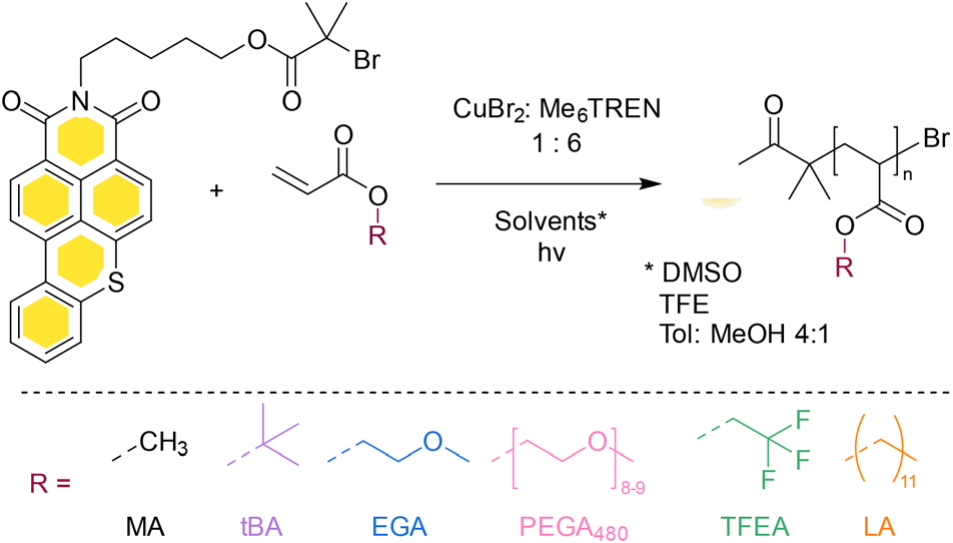
Reaction scheme of polymerising Host-BiB with different monomers and solvents.

To develop a simple, yet efficient platform that relies on the use of a broad-spectrum low energy light and omits the addition of external PCs, we envisioned a dual-functionality component that serves both as initiator and as the PC, for different wavelengths. Unlike previous work, the PC has a single efficient initiating site for simple, rapid linear polymerisation, with kinetics and control comparable to typical UV-induced photo Cu-RDRP. Herein, we report the use of Hostasol-BiB (Host-BiB), a fluorescent initiator used to synthesise a variety of well-defined polymers using lower energy and visible wavelengths *via* photo Cu-RDRP. Importantly, we show that with the use of Host-BiB six different wavelengths can be employed for the synthesis of a variety of polymers (hydrophobic, hydrophilic and semi-fluorinated) with different molecular weights as well as *in situ* chain extension, which alongside matrix-assisted laser desorption/ionization time-of-flight (MALDI-ToF-MS) studies, verify high end-group fidelity (Fig. 1).

## Results and discussion

**Fig. 2:**
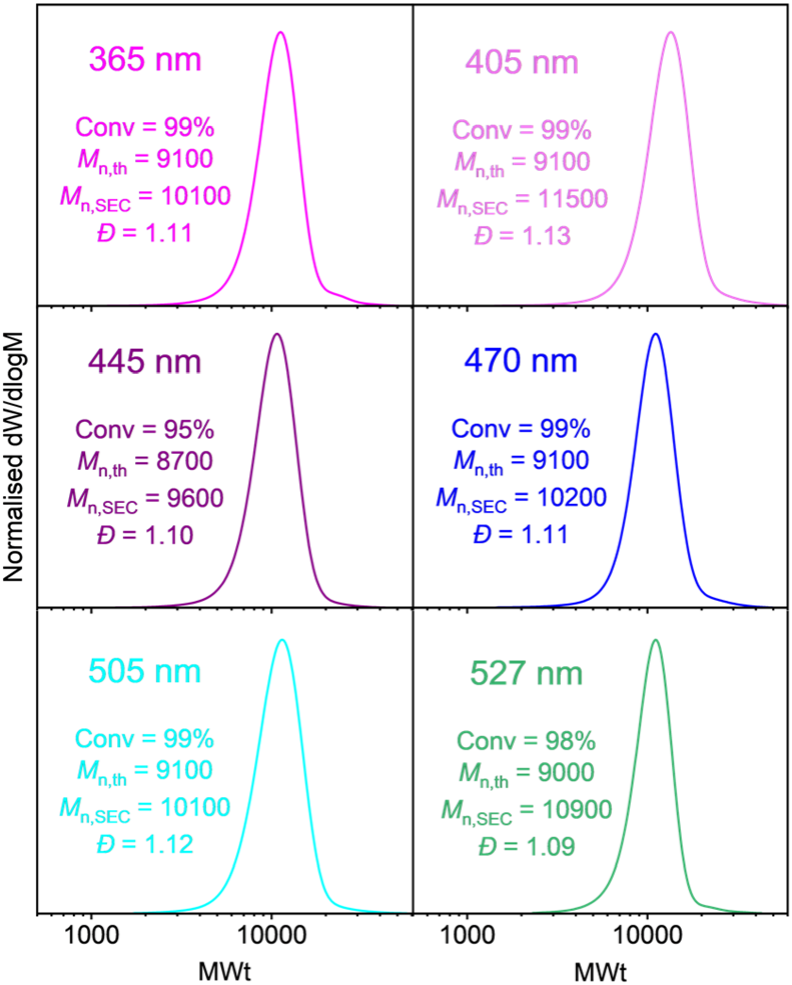
SEC traces of Host-MA_100_ synthesised with varying wavelengths. [MA] : [Host-BiB] : [CuBr_2_] : [Me_6_TREN] = [100] : [1] : [0.02] : [0.12] in DMSO (1 : 1, v/v) within 1 h.

Initially, we explored the photoinduced Cu-RDRP of methyl acrylate (MA) initiated with Host-BiB. As Host-BiB has an absorption maximum at *λ* = 460 nm (Fig. S4), our LED array with the closest output was chosen (*λ* ∼ 470 nm). The conditions used were [MA] : [Host-BiB] : [CuBr_2_] : [Me_6_TREN] = 100 : 1 : 0.02 : 0.12. After deoxygenation, reactions were exposed to LED irradiation with the temperature maintained at 5–10 °C (SI). After only 30 minutes under irradiation at 470 nm, 83% monomer conversion was achieved to give poly(methyl acrylate) (PMA) having low dispersity (*Đ* = 1.09) and good agreement between the theoretical and experimental MWts (Fig. S5). However, the recent literature on the disparity between absorption maxima and polymer conversion encouraged us to explore a variety of wavelengths, particularly those red-shifted to the absorption maxima including cyan (∼505 nm), green (∼527 nm) and red (∼630 nm). Pleasingly, controlled polymerisation occurred at all the wavelengths used (*λ* ∼ 365, 405, 445, 470, 505, 527 and 630 nm) except for 630 nm, where no polymerisation was observed, even after irradiation for 24 hours (Fig. 2).

Action plots compare the UV-Vis absorbance of a PC component, in this case Host-BiB, to the monomer conversions achieved at varying wavelengths using the PC at set time intervals.^26^ Inspired by the work from Barner-Kowollik and Blasco,^24^ we set out to map an action plot, synthesising PMA and sampling after 5 minutes with irradiation at 7 different wavelengths with similar irradiance (Tables S1 and S2). While other action plots have been established using a specialised laser which produce identical photon fluxes at varied excitation wavelengths,^25,26^ we employed simple, commercially available Lumidox® II 96-Well LED arrays and instead plotted conversion according to the reported power output, the corrected irradiance of each array (obtained by measuring the irradiance through a photometer, then considering the meter's responsivity at each wavelength to Individually calculate correction factors to normalise the wavelengths) and the calculated and normalised photon flux (Fig. S6 and 3). Interestingly in all plots, whether normalised or not, red-shifted action plots were produced, comparable to previous action plot studies.^24^ The wavelength that produced the highest monomer conversion after 5 minutes in all cases was 505 nm, which is red-shifted from the maxima by approximately Δ*λ* = 45 nm, while the lowest monomer conversion in all plots was 470 nm, which previously would have been unexpected considering the 470 nm LED array is the closest to the absorption maxima. Across all plots, 527 nm had the second highest monomer conversion, despite very little overlap of the Host-BiB with the green LED array's spectral distribution (Fig. S4). 365 nm also resulted in relatively high monomer conversion per photon flux, which could be partially due to direct excitation of free ligand and CuBr_2_/Me_6_TREN as utilised in typical photo Cu-RDRP. While Host-BiB under 365 nm resulted in 24% conversion, a reaction with EBiB and no Hostasol achieved 18% monomer conversion in 5 minutes, suggesting Hostasol is still participating in the energy transfer for polymerisation under 365 nm.

**Fig. 3:**
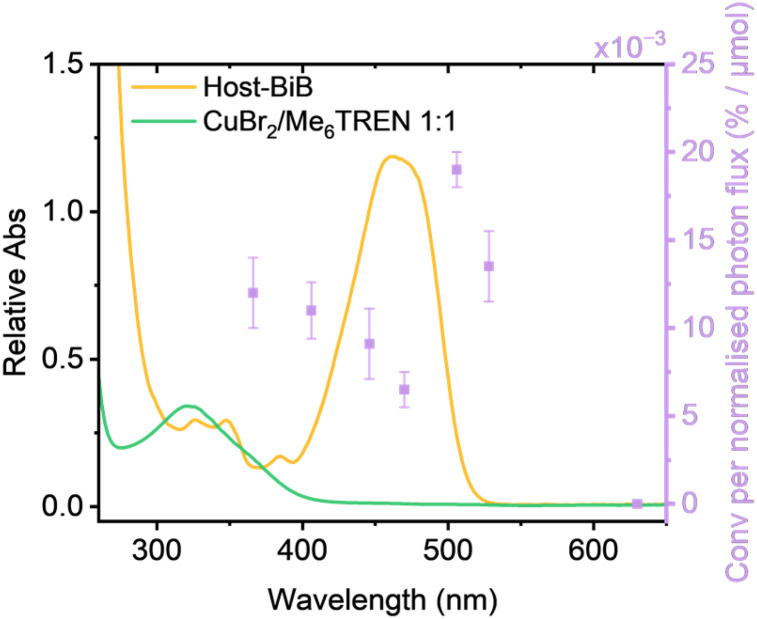
Wavelength-dependent polymerisation of MA by different wavelengths overlaid with the UV-Vis spectrum of Host-BiB in DMSO (orange line) and CuBr_2_/Me_6_TREN (green line), with purple squares showing % (conversion) per normalised photon flux (μmol) and error bars showing differences in % after repeats.

To elucidate the dual role of Host-BiB, as well as to rule out potential side reactions and probe other conditions (Table 1), a series of control experiments were performed. Firstly, reactions with no Me_6_TREN (ligand) resulted in no polymerisation whereas reactions with no Cu complex, no Cu species (Cu^II^Br_2_) or no initiator resulted in an uncontrolled polymerisation, giving products with broad molecular weight distributions. With no initiator, 21% conversion was achieved which is similar to reported photoexcited free ligand activity.^11^ Hostasol alcohol (Host-OH) was also examined as a PC with catalytic amounts similar to quantities used in recent literature, and one reaction with equal parts Host-OH to initiator, ethyl α-bromoisobutyrate (EBiB). Both reactions gave identically controlled polymers, confirming only small loadings of the PC is required. One control with EBiB and no Hostasol present resulted in no conversion under irradiation at 505 nm within an hour while a non-irradiated sample with Host-BiB produced no polymer after 24 hours. When the copper complex ([CuBr_2_/L]) was decreased to [CuBr_2_] : [L] = [0.005] : [0.03], the dispersity increased considerably to 1.38, with significant high molecular weight tailing ascribed to decreased deactivation (Fig. S10). When an equivalent ratio of copper and ligand was used, [CuBr_2_] : [L] = [0.02] : [0.02], no monomer conversion was achieved, which is expected due to previous studies establishing the need for excess ligand.^11,23^

**Table 1:** Control experiments under 505 nm for 1 h with DP_target_ = 100

Entry	[I] : [Cu^II^] : [L] : [PC]^a^	Conv^b^ (%)	*M*_n,SEC_ c	*Đ*
1	— : 0.02 : 0.12 : 1	21	42 100	2.19
2	1 : — : — : —	28	62 100	Bi-modal
3	1 : 0.02 : — : —	0	—	—
4	1 : — : 0.12 : —	>99	32 000	5.63
5^d^	1 : 0.02 : 0.12 : 0.005	98	10 300	1.08
6^d^	1 : 0.02 : 0.12 : 1	>99	9400	1.09
7	1 : 0.02 : 0.12 : —	>99	10 100	1.12
8^d^	1 : 0.02 : 0.12 : —	0	—	—
9	1 : 0.005 : 0.03 : —	80	9300	1.38
10	1 : 0.02 : 0.02 : —	0	—	—

a [MA] : [Host-BiB] : [CuBr_2_] : [Me_6_TREN] : [Host-OH] = 100 : *w* : *x* : *y* : *z* in 50% (v/v) DMSO.

b Determined from ^1^H NMR.

c Determined from THF SEC analysis.

d I = EBiB instead of Host-BiB.

**Fig. 4:**
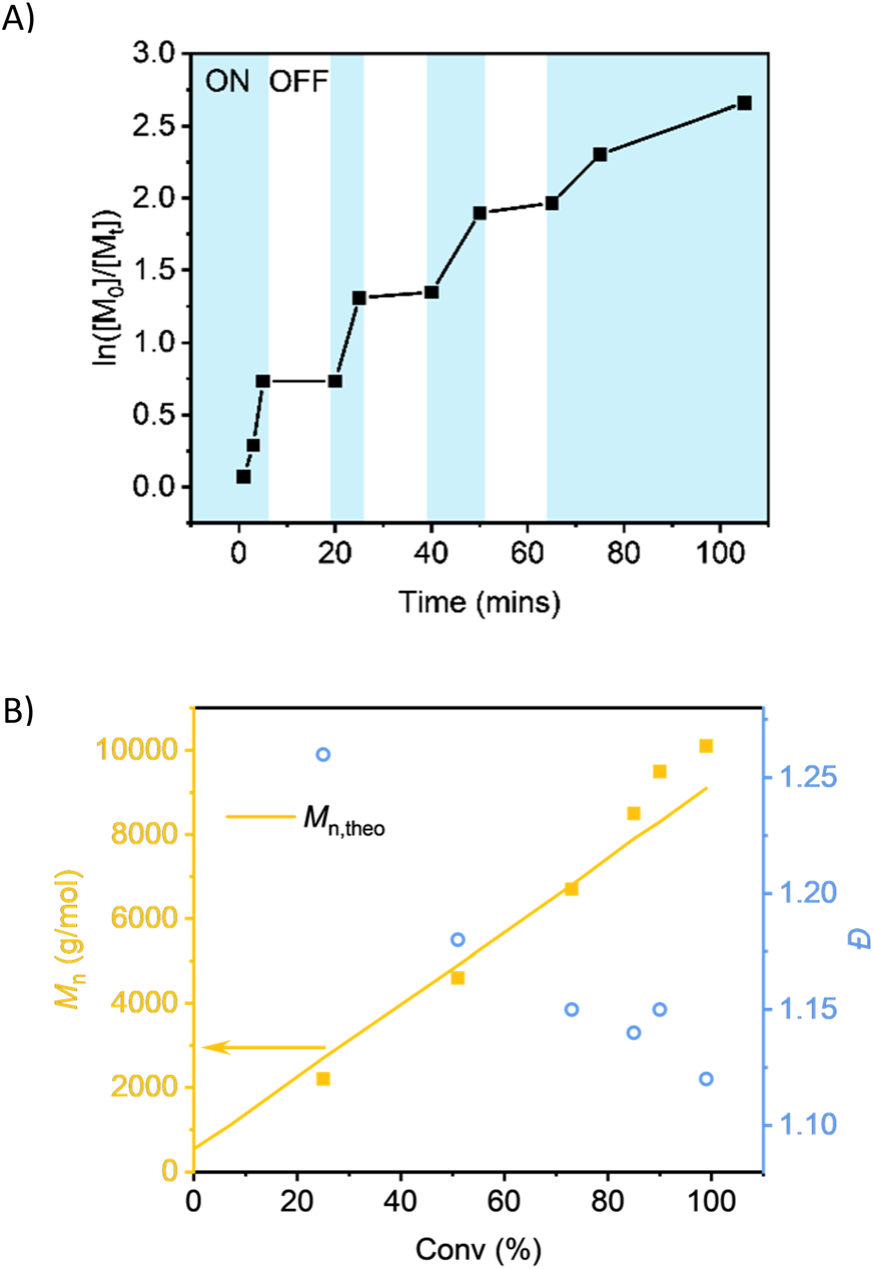
Polymerisation kinetics of Host-MA DP_target_ = 100 under 505 nm irradiation. (A) Evidence of temporal control with light on (blue) and off (white) (B) conversion *vs.* molecular weight and dispersity.

Polymerisation of MA under irradiation at 505 nm with Host-BiB was monitored by samples being taken at periodic intervals throughout the polymerisation (Fig. 4). The kinetic studies showed linear first order kinetics, demonstrating the controlled nature of the polymerisation with constant radical concentration. As anticipated, the experimental *M*_n_ (*M*_n,SEC_) increased linearly with conversion and stayed in good agreement with the theoretical values, as well as the dispersity remaining low at high conversion (*Đ* < 1.15). Temporal control was explored through “light on/light off” periods, showing negligible monomer conversion in the dark and upon further irradiation, the polymerisation continued.

Kinetics were tested at all 6 wavelengths. While the action plot indicated certain wavelengths polymerise faster than others within the first 5 minutes, >90% monomer conversion was reached within 1 hour for all wavelengths, showing good control (Fig. S13). Each wavelength resulted in relatively fast polymerisation rates compared to other photo Cu-RDRP studies, particularly recent work on utilising catalytic amounts of fluorescent PCs.^46^ A kinetic study of free Hostasol with EBiB was also compared to the polymerisation with Host-BiB and showed almost identical monomer conversion over time under 505 nm, signifying no difference in the polymerisation whether the PC is exploited as a dual initiator or not.

**Fig. 5:**
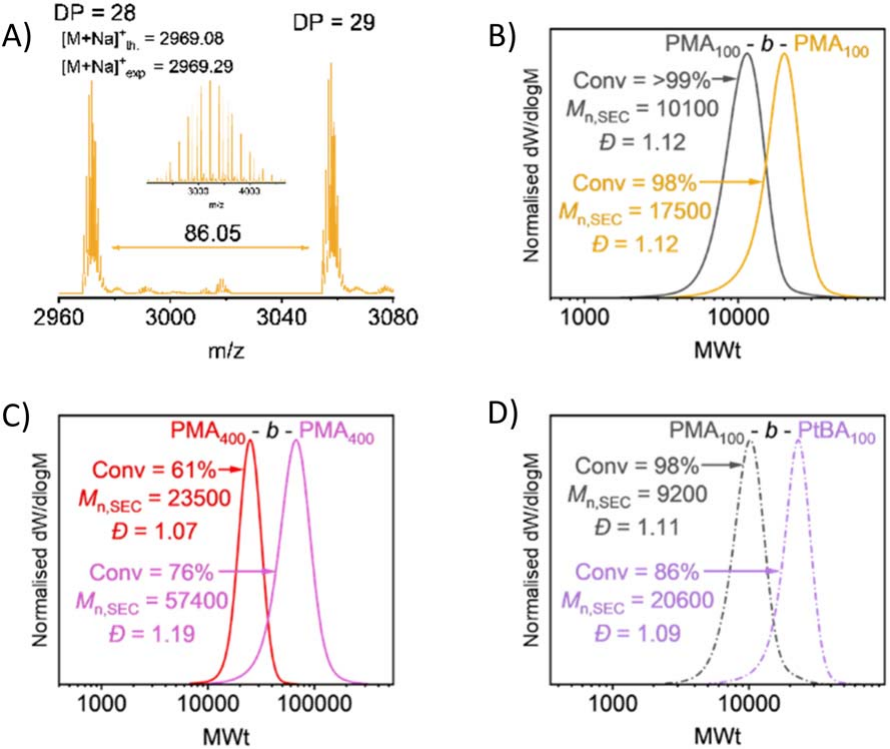
Evidence of high end-group fidelity with (A) MALDI-ToF-MS of PMA_25_ and *in situ* chain extensions showing (B) PMA_100_-MA_100_, (C) PMA_400_-MA_400_, and (D) PMA_100_-*t*BA_100,_ with all polymers synthesised under 505 nm irradiation either in 1 h (A–C) or 24 h (D).

Chain extension experiments were performed to confirm end-group fidelity and control. Firstly, PMA_100_ was targeted reaching full conversion and then an aliquot of MA was added *in situ* to form PMA_100_-*b*-MA_100_. The increase in molecular weight (Fig. 5), demonstrates the polymerisation's pseudo-‘living’ character. This was then repeated for blocks of PMA with DP = 400, and copolymers of PMA_100_-*b-t*BA_100_, which again displayed clear shifts towards higher molecular weight. To further prove the retention of the Hostasol α-end group as well as a high fidelity in the bromine ω-end group, MALDI-ToF was conducted on PMA_25_, showing a distinct singular distribution with characteristic isotopic splitting of bromine (Fig. 5A and S16).

**Fig. 6:**
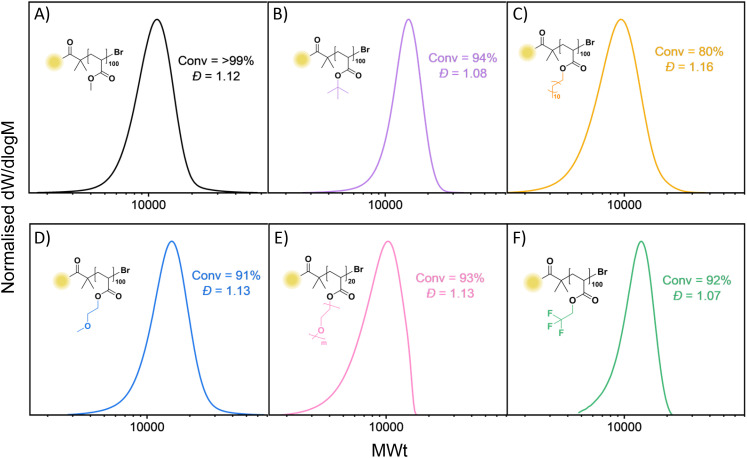
SEC traces of different monomers polymerised under 505 nm irradiation for 1 h (A, D and E) or 24 h (B, C and F).

To examine the versatility of our approach, higher molecular weights were targeted. Up to DP_target_ = 1600, low dispersity (*Đ* = 1.14) was retained within 1 hour with 71% monomer conversion. DP_target_ = 2500 required only 2 hours to achieve similar conversion, with *Đ* = 1.22, while DP_target_ = 4000 resulted in loss of control (*Đ* = 1.83) with tailing at lower MWts and lower monomer conversion (65%) (Table 2 and Fig. S17). To optimise our system, DP_target_ = 4000 was repeated with more solvent to investigate whether a less viscous reaction could be better controlled. Despite higher monomer conversion (90%), even lower MWt and earlier termination was observed (*Đ* = 1.50, Fig. S18) (Fig. 6).

**Table 2:** Polymerisation of MA with different DP targets under 505 nm

DP^a^	Conv^b^ (%)	Time (hrs)	*M*_n,SEC_ c	*Đ*
400	61	1	23 500	1.07
800	65	1	55 200	1.10
1600	71	1	104 800	1.14
2500	71	2	135 600	1.22
4000	65	2	424 300	1.83

a [MA] : [Host-BiB] : [CuBr_2_] : [Me_6_TREN] = *x* : 1 : 0.02 : 0.12 in 50% (v/v) DMSO.

b Determined from ^1^H NMR.

c Determined from THF SEC analysis.

**Fig. 7:**
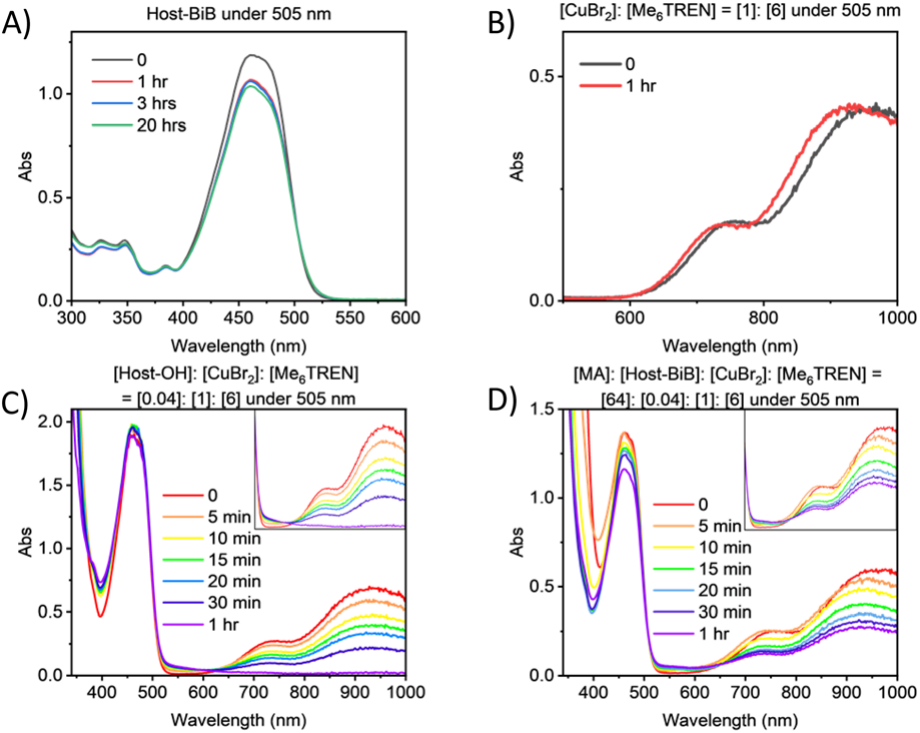
Time-dependent UV-Vis spectra and kinetic profiles of (A) Host-BiB, (B) CuBr_2_/Me_6_TREN, (C) Host-OH and CuBr_2_/Me_6_TREN and (D) MA, Host-BiB and CuBr_2_/Me_6_TREN, in DMSO under irradiation at 505 nm.

To expand the scope of this study, other acrylic monomers were investigated (Fig. 7, S16 and Table S3). Ethylene glycol methyl ether acrylate (EGA) and poly(ethylene glycol methyl ether acrylate) (PEGA_480_) were employed as hydrophilic monomers, with their polymerisation yielding high monomer conversions (91% and 93%, respectively with *Đ* = 1.13 each) within as little as 1 hour. More hydrophobic monomers such as *tert*-butyl acrylate (*t*-BA) and lauryl acrylate (LA) which are known to phase separate in DMSO upon polymerisation,^64^ were polymerised using toluene: methanol 4 : 1 (Tol : MeOH) and trifluoroethanol (TFE) as solvents under irradiation at 505 nm. While both solvents resulted in slower polymerisations than those in DMSO, PtBA reached 94% monomer conversion in 24 hours with low dispersity (*Đ* = 1.13) in TFE while Tol : MeOH resulted in a lower conversion and slightly higher dispersity (70% and *Đ* = 1.16). PLA in TFE reached full monomer conversion, however phase separation occurred while no phase separation occurred in Tol : MeOH 4 : 1, resulting in lower dispersity (*Đ* = 1.16) but lower conversion (80%). 2,2,2-Trifluoroethyl acrylate (TFEA) was trialled as a semi-fluorinated monomer and produced well-defined polymers in TFE (92% conversion and *Đ* = 1.07).

Photophysical data was collected to probe the mechanistic behaviour of the PC, namely ultraviolet-visible spectroscopy (UV-Vis), steady-state fluorescence, time-resolved fluorescence/time-correlated single-photon counting (TCSPC) and cyclic voltammetry (CV). Detailed mechanisms have been previously proposed for visible light photo Cu-RDRP without an embedded PC.^23,61^ UV-Vis measurements followed the behaviour after irradiation over time under 505 nm. Firstly, the photostability of Hostasol was explored, with the lack of change in the peaks verifying Hostasol's capacity to withstand photo-degradation, unlike certain organic dyes previously mentioned (Fig. 7A). No change was then observed for Cu^II^Br_2_/Me_6_TREN within 20 hours as expected (Fig. 7B), while when Host-OH was added the absorbance maxima (*λ*_max_ = 950 nm and *λ* = 750 nm) attributed to absorption *via* the d–d transitions of the Cu^II^ complex reduced drastically within 1 hour, signifying the photoreduction of Cu^II^ therefore the generation of active species (Fig. 7C). When a solution mimicking the polymerisation reaction was monitored by UV-Vis (Fig. 7D), a smooth reduction of the Cu^II^ species was observed and the Host-BiB peak showed decreasing absorption, indicating a permanent change, due to the growth of polymer chains bearing the Host moiety as the α-end group.

From the steady-state fluorescence, the quantum yield of Host-OH was also measured, validating its photocatalytic capacity with a high fluorescence quantum yield, *Φ*_F_ = 0.91 (Table S4). Quenching studies, measured using TCSPC, were used to determine the interaction of Cu^II^Br_2_/Me_6_TREN and free Me_6_TREN with the PC throughout the polymerisation. Following work by Hu and colleagues,^48^ a preference for a reductive or oxidative pathway for different PCs could be proposed depending on the rate of decrease of fluorescence decay with the increase of the copper ligand complex or only free ligand.

**Fig. 8:**
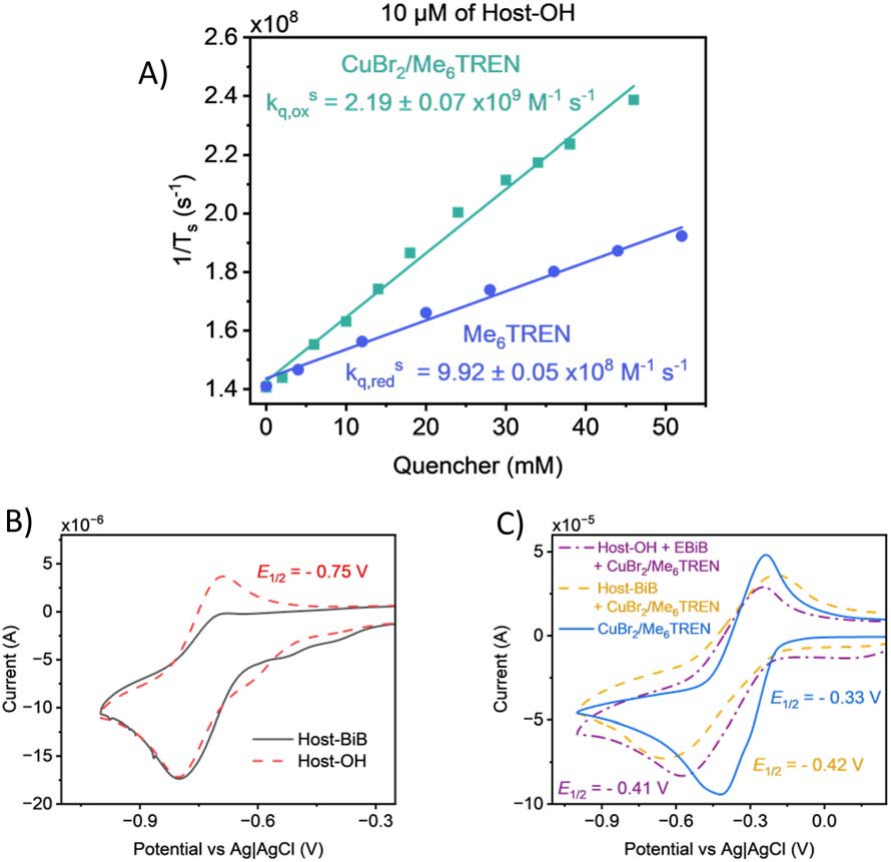
(A) Quenching study plot of the inverse fluorescence lifetime of Host-OH through TCSPC with *λ*_ex_ = 455 nm, using Cu^II^Br_2_/Me_6_TREN (1 : 1) (green) *vs.* Me_6_TREN. (B) Host-OH (red) *vs.* Host-BiB (black) and (C) Cu^II^Br_2_/Me_6_TREN (blue), Host-BiB and Cu^II^Br_2_/Me_6_TREN (orange) and Host-OH, EBiB and Cu^II^Br_2_/Me_6_TREN (purple) with 0.001–0.01 M concentration, in a 0.1 M tetrabutylammonium hexafluorophosphate solution in DMSO with scan rate 0.1 V s^−1^*vs.* Ag/AgCl.

When comparing the decrease in the decay of Host-OH with additional quencher, Cu^II^Br_2_/Me_6_TREN (1 : 1) had a rate constant of 2.19 ± 0.07 × 10^9^ M^−1^ s^−1^ while Me_6_TREN was 9.92 ± 0.05 × 10^8^ M^−1^ s^−1^ (Fig. 8A). Therefore, a 69.4% preference for the reductive pathway was calculated when considering five times more free ligand is used than Cu^II^Br_2_/Me_6_TREN (1 : 1). Therefore the formation of the PC as a radical anion is more likely before the reduction of Cu^II^ to Cu^I^ instead of the oxidative pathway where the PC forms a radical cation before metal reduction.^23^ An equivalent preference for a reductive pathway was found when Host-BiB was quenched (69.7%) (Fig. S21), suggesting the same mechanism is followed whether Hostasol is used only as a PC or dual PC initiator (Fig. S22). This was expected after our kinetic experiments showed the same rate of polymerisation for free Hostasol systems and for PC-initiated reactions. Despite forming a radical, an intramolecular energy transfer to the C–Br bond for Host-BiB is kinetically and spatially unfavourable, which is also demonstrated by the uncontrolled reaction with Host-BiB and no Cu^II^Br_2_/Me_6_TREN mentioned previously. It was also deduced that the PC only excited to a singlet state and did not participate in intersystem crossing into a triplet, as no change in the decay was observed whether conducted in open air or an inert atmosphere.

CV studies were carried out to provide information about the redox properties of Hostasol as a PC and dual PC initiator and how it differs with different components used in the polymerisation. The voltammogram of Host-OH displayed a clear reductive and corresponding oxidative peak with a negative half-wave potential (*E*_1/2_) of −0.75 V. Host-BiB, however, loses the oxidative peak, indicating the irreversible formation of a radical species (Fig. 8B). Since the reductive peak remains identical between Host-OH and Host-BiB, the ability of the PC is the same whether acting as a dual initiator or not, as found during our experiments and TCSPC. A CV of only Cu^II^Br_2_/Me_6_TREN displays reversible behaviour with a *E*_1/2_ of −0.33 V, as expected.^65^ When Host-OH and EBiB were introduced, quasi-reversible behaviour was observed with a new *E*_1/2_ of −0.41 V and with an enhanced cathodic scan expected of rapid initiator activation with a well-known initiator (Fig. 8D). When Host-BiB was added instead, an equivalent change in the scan was measured with a similar *E*_1/2_ to Host-OH and EBiB (−0.42 V), reaffirming Host-BiB as an effective initiator directly comparable with EBiB.

## Conclusion

In summary, we report the use of Host-BiB as a simultaneous PC and initiator for the fast, yet controlled synthesis of polyacrylates through photo Cu-RDRP, using a range of different wavelengths. By exploiting the dual role of this fluorescent dye, excellent control over the polymerisation is achieved, generating well-defined polymers with high monomer conversion and end-group fidelity, thus providing access to *in situ* chain extensions and block copolymers, whilst achieving high molecular weights. An action plot revealed high activity when red-shifted, supporting a mismatched reactivity and absorption phenomenon, while control experiments provide an insight into the initiating role of this PC. We anticipate that our approach will serve as a facile, yet versatile platform for photo Cu-RDRP of acrylates at various wavelengths, without the need for externally added PCs.

## Author contributions

M. D. H: investigation; methodology; formal analysis; visualisation; validation; writing – original draft. B. Z: investigation. E. L: supervision; writing – review and editing. T. J: supervision; writing – review and editing. D. H: conceptualisation; funding acquisition; methodology; project administration; resources; supervision; writing – review and editing.

## Conflicts of interest

There are no conflicts to declare.

## Supplementary Material

SC-OLF-D5SC05171A-s001

## Data Availability

The data supporting this article have been included as part of the SI. Supplementary information is available. See DOI: https://doi.org/10.1039/d5sc05171a.
