# Supplementary material for: Visible-light-induced copper-mediated reversible deactivation radical polymerisation without additional photocatalysts
Source: Chem Sci. 2025 Aug 28;16(37):17436–43. doi: 10.1039/d5sc05171a (PMC12403980; doi:10.1039/d5sc05171a)
Supplement: SC-016-D5SC05171A-s001 [file SC-016-D5SC05171A-s001.pdf]

Supplementary Information (SI) for Chemical Science.
This journal is © The Royal Society of Chemistry 2025

Supporting Information

Visible-Light-Induced Copper-Mediated Reversible Deactivation Radical Polymerisation Without Additional Photocatalysts

Mia D. Hall,[ab] Evelina Liarou,[a] Boyu Zhao,[a] Tanja Junkers,[b*] and David Haddleton[a*]

[a.] University of Warwick, Department of Chemistry, Library Road, Coventry, CV4 7AL, UK. Email: d.m.haddleton@warwick.ac.uk

[b.] Polymer Reaction Design Group, School of Chemistry, Monash University, 19 Rainforest Walk, Clayton, VIC 3800, Australia. E-mail: tanja.junkers@monash.edu

Experimental

Materials

Methyl acrylate (MA, 99%), tert-butyl acrylate (t-BA, 98%), ethylene glycol methyl ether acrylate (EGA, 98%), poly(ethylene glycol) methyl ethyl acrylate ($PEGA_{480}$, 97%), %), ethyl α-bromoisobutyrate (EBiB, 98%), copper(II) bromide ($Cu(II)Br_2$, 99%) and all the solvents were purchased from Sigma-Aldrich and used as received. Lauryl acrylate (LA, 90%) was purchased from Sigma-Aldrich and passed through a plug of basic alumina immediately before use. 2,2,2-Trifluoroethyl acrylate (TFEA, 99%) was purchased from Apollo Scientific and used as received. "Hostasol" (thioxantheno[2,1,9-dej]isochromene-1,3- dione) was supplied by Clariant and Hostasol alcohol (Host-OH) 2-(8-hydroxy-3,6-dioxaoctyl)thioxantheno[2,1,9-dej]isoqiunoline-1,3-dione was synthesised according to the literature and stored in the dark prior to use.[1] Tris-(2-(dimethylamino)ethyl)amine (Me_6Tren) was synthesised according to the literature and stored at 4°C prior to use.[2]

Instrumentation

^{1}H Nuclear Magnetic Resonance. ^{1}H NMR spectra were recorded on Bruker DPX-300 or DPX400 spectrometers, using samples dissolved in deuterated chloroform ($CDCl_3$) obtained from Sigma-Aldrich. Chemical shifts are given as δ in ppm downfield from the internal standard tetramethylsilane (TMS) δ = 0 ppm. Monomer conversions were calculated via ^{1}H NMR spectroscopy by comparing the integrals of monomeric vinyl protons to polymer signals. All spectra were analysed using ACD/NMR processor.

Size Exclusion Chromatography. SEC measurements were carried out on an Agilent Infinity II MDS instrument with differential refractive index (DRI), viscometry (VS), dual angle light scatter (LS) and dual wavelength UV detectors. The system was equipped with 2 x PLgel Mixed C columns (300 x 7.5 mm) and a PLgel 5 µm guard column. The eluent used was THF with 0.01 % butylated hydroxytoluene (BHT) as an additive. Samples were run at 1 mL/min at 30°C and were filtered through a PTFE membrane with a pore size of 0.2 µm before injection. A conventional calibration was made using narrow molecular weight poly(methyl methacrylate) (11

standards between 2,210,000- 1010 Da) and polystyrene (12 standards between 364,000-160 Da) from Agilent EasiVials. Experimental molar mass ($M_{n,SEC}$) and dispersity (Đ) values of synthesised polymers were determined using the DRI trace and by this calibration using Agilent GPC/SEC software.

Matrix-assisted laser desorption/ionization time-of-flight (MALDI-ToF-MS). Polymer samples and poly(ethylene glycol) (PEG) calibrants were prepared in THF at a concentration of 10 mg/mL, with 1 mg/mL of NaI as the cationising agent. These samples were then mixed at a ratio of 1:1 with a 40 mg/mL solution of trans-2-[3-(4-tert-butylphenyl)-2-methyl-2-propenylidene] malononitrile (DCTB) in THF. 0.5 µL of each sample was then applied to the MTP 384 ground steel target plate and analysed using either a Bruker Autoflex or Ultraflex II ToF/ToF Analyser, with a nitrogen laser delivering 2 ns laser pulses at 337 nm with positive ion ToF detection using an accelerating voltage of 25 kV.

UV-Vis spectroscopy. UV-Vis spectra were recorded on an Agilent Technologies Cary 60 UV-Vis spectrometer in the range of 200-1100 nm using a quartz cuvette (purchased from Starna) with 10 mm optical length. For kinetics, Hostasol-BiB or Hostasol-OH (0.1 mg) with Cu(II)Br_2 (1 mg) and Me_6TREN (7.2 µL) was added to 3.6 mL DMSO and degassed for 15 minutes before getting placed in the LED set-up and irradiated with λ_{max}~ 505 nm. For quantum yield, the concentration of Hostasol-OH or rhodamine 6G in DMSO was varied for four measurements where λ_{max}~ 0.01 to 0.1.

Steady-state fluorescence spectroscopy. Fluorescence spectra were recorded on an Agilent Technologies Cary Eclipse Fluorescence Spectrophotometer. Samples with λ_{max}~ 0.01 to 0.1 recorded on UV-Vis were then ran on fluorescence. For emission scans, the excitation wavelengths for the different samples was set at λ = 460 nm (Hostasol-OH) and at λ = 537 nm (rhodamine 6G) with 2.5 nm slits.

Time-resolved fluorescence spectroscopy. Fluorescence decay spectra were recorded on a Horiba Fluorolog® fluorescence spectrophotomer, using a 455 nm NanoLED as the excitation light source, with fluorescence decay monitored around 555 nm with 950 volts over a 50 ns measurement range. Measurements were completed after a peak count of 1000. Spectra were measured in a 10 mm optical length quartz cuvette, with all samples prepared with concentrations of 10 µm and dissolved in DMSO. Quenching solutions were conducted by adding quencher solutions after each measurement between 2 – 50 mM. Blank traces were collected, referred to as a prompt, to establish the instrument response. The lifetime values were determined by fitting the time-resolved emission traces with an exponential decay combined with a Gaussian function to consider the instrument response.

Cyclic voltammetry. Cyclic voltammetry measurements were recorded on a CH-Instruments 600 E potentiostat connected to a PC running CHI600E Electrochemical Analyser software, using a 3 mm glassy carbon disc working electrode, a platinum wire coil as a counter electrode and silver/silver chloride wire as a reference electrode. The working and reference electrodes were polished with 0.05 µm alumina powder and rinsed sequentially with acetone, ethanol and MilliQ water. The silver wire was then placed into a glass capillary tube fitted with a Vycor® frit and filled with 3 M potassium chloride solution. The supporting electrolyte tetrabutylammonium hexafluorophosphate (0.217 g, 1 mmol) and DMSO (10 mL) were mixed and used as

background electrolyte. A background CV was recorded to confirm the absence of impurities and oxygen. Measurements using Host-OH, Host-BiB or EBiB were made at a concentration of 0.001 M while $CuBr_2/Me_6TREN$ had a concentration of 0.01 M. Measurements were then conducted with a scan rate of 0.1 V/s going from 0.6 V to – 1V in open air.

IR. FT-IR measurements were performed on a Shimadzu Spirit IR equipped with a SpecAc Golden Gate ATR. The wavelength used was from 500 to 4000 cm^{-1}, with 40 scans, at 4 cm^{-1} resolution.

LED irradiation set-up. Lumidox® II 96-Well LED arrays were purchased from Analytical Sales. The 96-position arrays with diffuse mat and solid base were set at the powers specified. The same set-up as described in a previous paper was used, with the addition of a cooled steel plate to keep the set-up at ambient temperature, checked by a RS PRO RS-172 Temperature & Humidity Data Logger.[3] Irradiance (mW cm^{-2}) output of the arrays was measured over the top of an individual sample hole in the gold plate with two LED wells visible as used in the polymerisation reaction, using a ThorLabs S142C Integrating Sphere Photodiode Power Sensor with Silicon Detector, connected to a ThorLabs PM400 optical power meter at the emission wavelength as specified by the LED supplier.

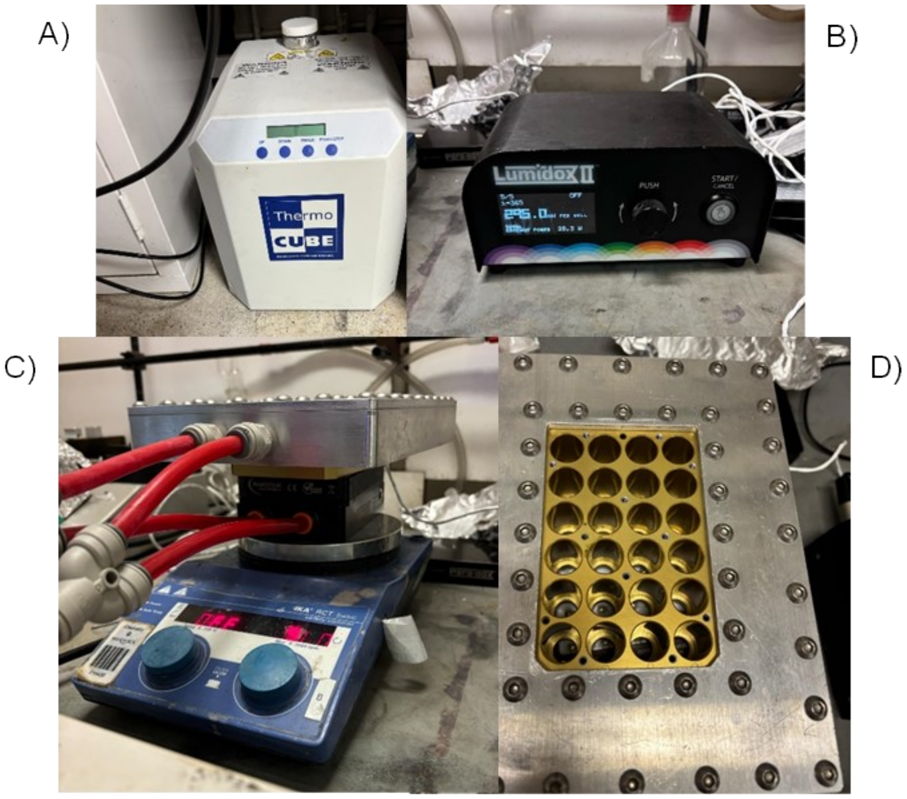

Figure S1: Images of LED cooling set-up with A) Chilling box B) LED controller C) Stacked plates and LED D) Zoom-in on gold plate inside cooled steel plate.

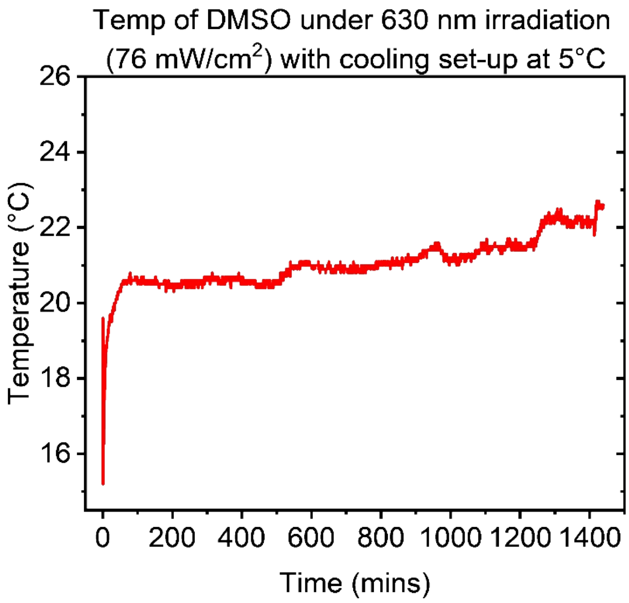

Figure S2: Temperature monitoring of DMSO under λ = 630 nm irradiation over one day using a temperature logger.

Table S1: Data for the different wavelengths provided by Lumidox® II LED Arrays and the use of a Thorlabs' power meter to measure irradiance and then calculate photonflux, considering each wavelength's

Wavelength (nm)	mW per well [a]	Responsivity ($x10^{-5}$ A/W) [b]	Correction factor [c]	Measured irradiance (mW cm^{-2})	Corrected irradiance (mW cm^{-2})	Photonflux (µmol) [d]	Normalised photonflux (µmol)
365	195	4.54	5.11	13	66	397	2014
405	175	7.51	3.09	22	68	745	2302
445	235	12.7	1.83	42	77	1562	2865
470	220	16.1	1.44	57	82	2240	3222
505	175	20.6	1.13	58	66	2449	2786
527	180	23.2	1	59	59	2600	2600
630	305	36.3	0.64	240	154	12640	8111

responsivity.

[a] Reported power output by Analytical Sales and Services, Inc.[4] [b] Reported responsivity data by Thorlabs for the S142C photodiode power sensor.[5] [c]

$$Correction\ factor\ for\ a = \frac{Responsivity\ at\ one\ wavelength\ (e.g.\ 527\ nm)}{Responsivity\ at\ a}$$ [d]

$$Number\ of\ photons\ s^{-1}m^{-2}(NP) = \frac{I \times \lambda \times 10^{-9}}{h \times c}\ then\ Photon\ flux = \frac{NP}{NA \times 10^{-6}}$$ where I is irradiance, λ is the wavelength, h is Planck's constant, c is the speed of light, NA is Avogadro's constant.

Experimental procedures

Host-BiB synthesis

2 g Hostasol-OH along with 3.6 mL triethylamine was added to 80 mL chloroform, which was cooled to 0°C and kept under nitrogen. 3.1 mL 2-bromo-isobutyryl bromide was added into 20 ml chloroform and added dropwise into the flask over an hour. The mixture was then stirred and reacted overnight at room temperature. After cooling to 0°C, 10 mL of methanol was added to remove excess 2-bromoisobutyryl bromide and left to stir at room temperature for an additional hour. The crude product was extracted with 1 M hydrochloride (HCl) aqueous solution (3 x 100 ml), saturated sodium hydrogen carbonate ($NaHCO_3$) aqueous solution (3 x 100 ml) and then dried with magnesium sulphate ($MgSO_4$). The crude product was dissolved in 40 ml chloroform, and 160 ml hexane was slowly added. The mixture was stored in the fridge overnight where the orange initiator crashed out. Hostasol-BiB was analysed by ^{1}H NMR and ESI-MS.

^{1}H NMR (400 MHz, $CDCl_3$, ppm): δ = 8.62 (d, 1H, J = 12 Hz), 8.42 (d, 1H, J= 12 Hz), 8.22 – 8.17 (m, 2H), 7.51 (d, 1H, J = 12 Hz), 7.43 – 7.36 (m, 3H), 4.22 (t, 4H, J = 8 Hz), 1.92 (s, 6H), 1.86 – 1.75 (m, 4H), 1.59 – 1.51 (m, 2H). ESI-MS m/z: calcd for $C_{27}H_{24}NO_4SBr$ 560.05 $[M+Na]^+$, observed 560.0.

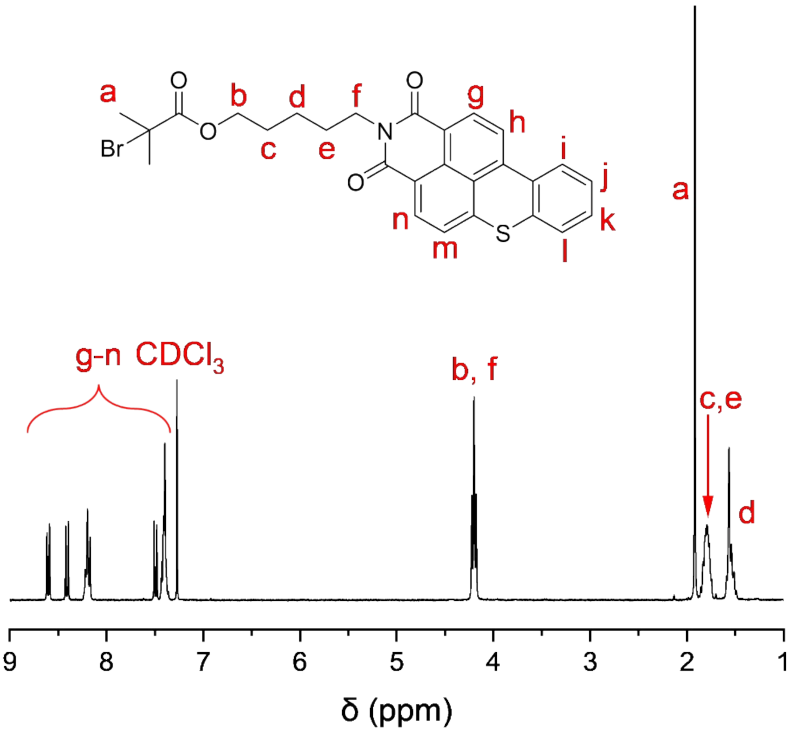

Figure S3: ^{1}H NMR (400 MHz) of Host-BiB in $CDCl_3$.

General procedure for the photo-induced polymerisation of MA DP_{target} = 100 with Host-BiB as a PC initiator

MA (0.5 mL, 5.55 mmol, 100 eq), Host-BiB (29.75 mg, 55.4 µmol, 1 eq), $CuBr_2$ (0.25 mg, 1.12 µmol, 0.02 eq), Me_6Tren (0.45 µL, 6.73 µmol, 0.12 eq) and DMSO or TFE (0.5 mL) were added to a septum sealed vial with a magnetic stirrer and deoxygenated by purging with nitrogen for 12 min. Polymerisation commenced upon addition of the deoxygenated reaction mixture to the LED set-up. Samples were taken at pre-defined intervals and conversions were measured using ^{1}H NMR and passed through an alumina column to remove traces of remaining copper before SEC analysis.

General procedure for in-situ chain extensions

Firstly, the procedure for polymerisation of MA above was followed. After 90 min, 0.5 ml of MA or Tba (100 eq) and DMSO were added to the mixture and the solution was deoxygenated by sparging with nitrogen for 12 min. Samples were taken at pre-defined intervals and conversions were measured using ^{1}H NMR and passed through an alumina column before SEC analysis.

General procedure for the photo-induced polymerisation of MA_{100} with Host-OH as PC only

MA (0.5 mL, 5.55 mmol, 100 eq), EBiB (8.16 µL, 55.4 µmol, 1 eq), Host-OH (29.75 mg, 55.4 µmol, 1 eq) or (0.15 mg, 279.3 nmol, 0.005 eq), $CuBr_2$ (0.25 mg, 1.12 µmol, 0.02 eq), Me_6Tren (0.45 µL, 6.73 µmol, 0.12 eq) and DMSO or TFE (0.5 mL) were added to a septum sealed vial with a magnetic stirrer and deoxygenated by purging with nitrogen for 12 min. Polymerisation commenced upon addition of the degassed reaction mixture to the LED set-up. Samples were taken at pre-defined intervals and conversions were measured using ^{1}H NMR and passed through an alumina column before SEC analysis.

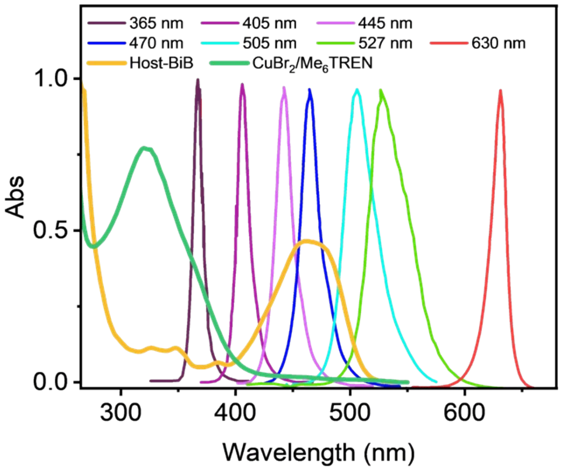

Figure S4: Normalised spectral distributions of the seven different Lumidox® II 96-Well LED arrays used, overlaid with the normalised UV-Vis of Host-BiB (thicker orange line) and $CuBr_2$/Me_6TREN (thicker green line). Distributions provided by Analytical Sales and Services, Inc.[4]

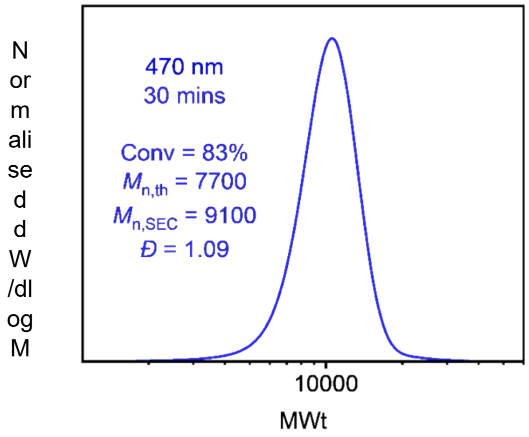

Figure S5: SEC trace of Host-PMA DP_{target} = 100, synthesised by photo Cu-RDRP using λ ~ 470 nm for 30 min.

Table S2: Photo Cu-RDRP of MA within 5 minutes using a variety of different wavelengths with DP_{target} = 100.[a]

Wavelength (nm)	Conv (%)[b]	$M_{n,th}$ (g/mol)	$M_{n,SEC}$[c]	$Đ$
365	24	2600	2900	1.19
405	25	2700	2700	1.21
445	26	2800	3200	1.18
470	21	2300	2000	1.24
505	51	4900	4600	1.18
527	35	3500	3500	1.18
630	0	-	-	-

[a] [MA]: [Host-BiB]: [$CuBr_2$]: [Me_6TREN] = 100: 1: 0.02: 0.12 in 50% (v/v) DMSO. [b] Determined from ^{1}H NMR. [c] Determined from THF SEC analysis.

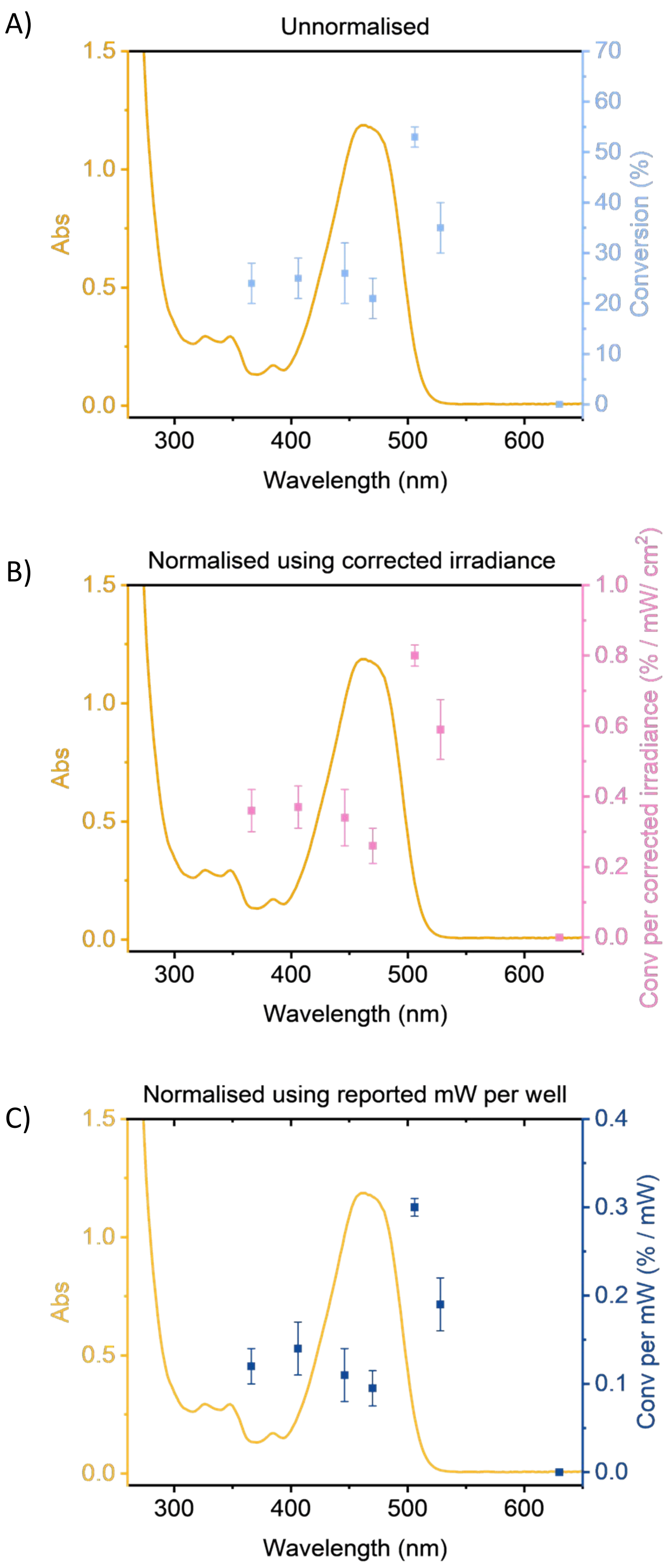

Figure S6: Wavelength-dependent polymerisation of MA by different wavelengths, overlaid with the UV-Vis spectrum of Host-BiB in DMSO (orange line). A) Shows unnormalised action plot with actual conversion data, where light blue squares show % (conversion) and error bars show difference following repeats. B) The same as A) except % is shown in pink and has been normalised with corrected irradiance to fairly compare wavelengths. C) Is the same as B) except % is shown in dark blue and normalised against the reported mW per well of the LED array (two wells used per sample).

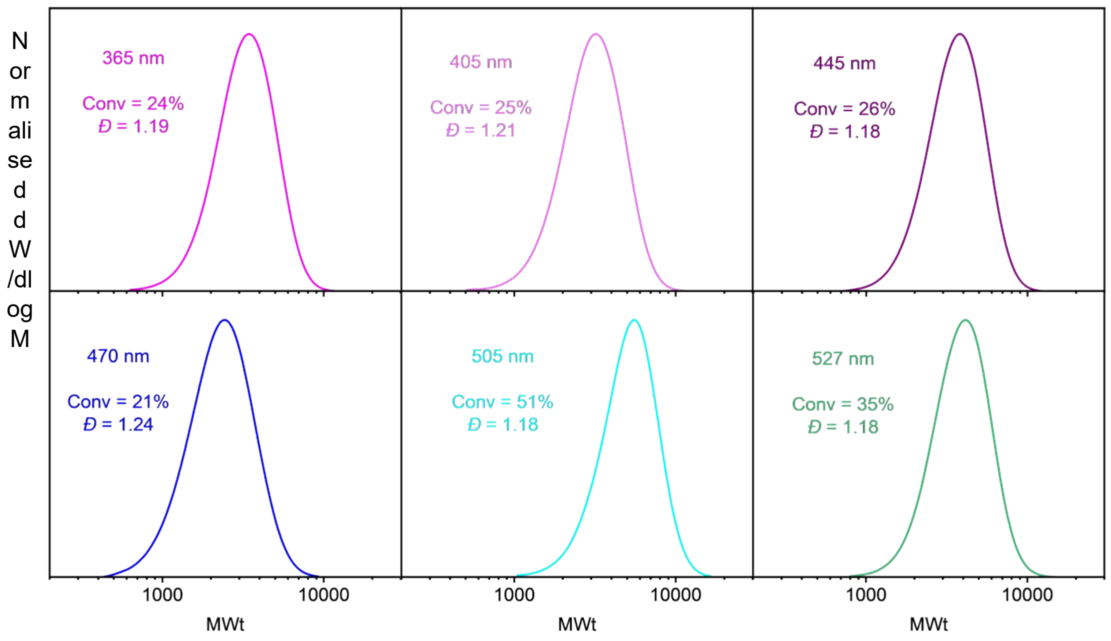

Figure S7: SEC traces of Host-PMA DP_{target} = 100, synthesised by photo Cu-RDRP using 6 different wavelengths (λ ~ 365, 405, 445, 470 and 527 nm) within 5 minutes.

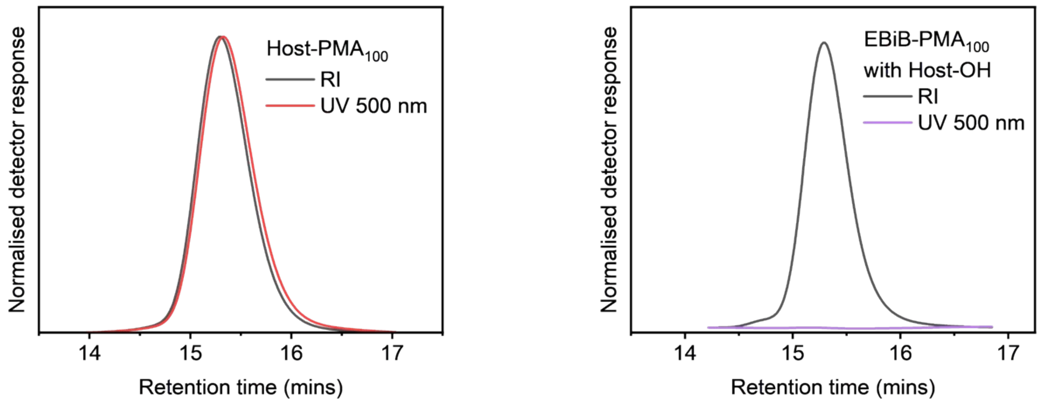

Figure S8: SEC traces comparing RI and UV 500 nm detectors against retention time for PMA DP_{target} = 100, synthesised by photo Cu-RDRP using λ ~ 505 nm with Host-BiB (left) and EBiB with Host-OH (right).

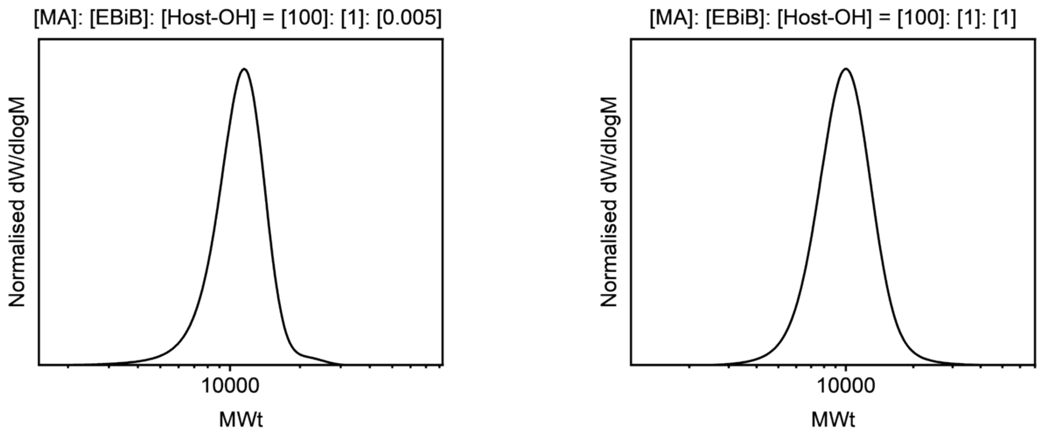

Figure S9: SEC traces of PMA DP_{target} = 100 with A) Host-OH used as a catalytic amount and B) Host-OH used at the same ratio of initiator, synthesised by photo Cu-RDRP using λ ~ 505 nm with Host-BiB (left) and EBiB with Host-OH (right).

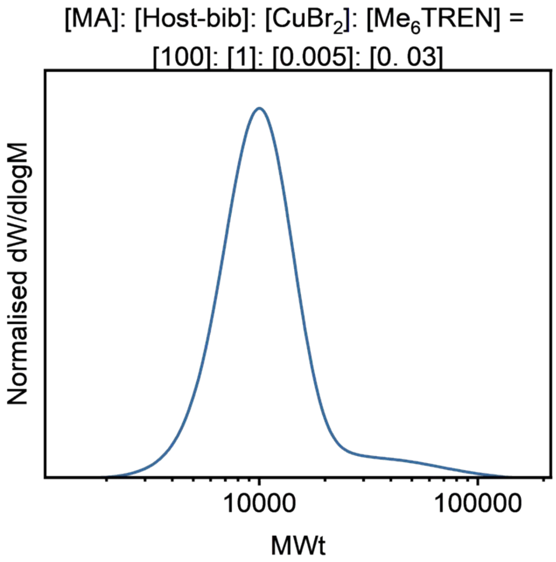

Figure S10: SEC trace of PMA DP_{target} = 100 with reduced amount of copper complex, synthesised by photo Cu-RDRP using λ ~ 505 nm.

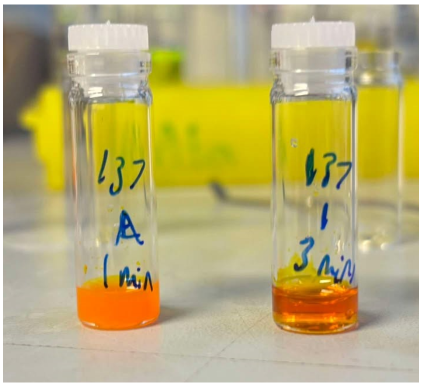

Figure S11: Image depicting the polymerisation of MA under irradiation at 505 nm with Host-BiB at 1 min (left vial) and 3 min (right vial).

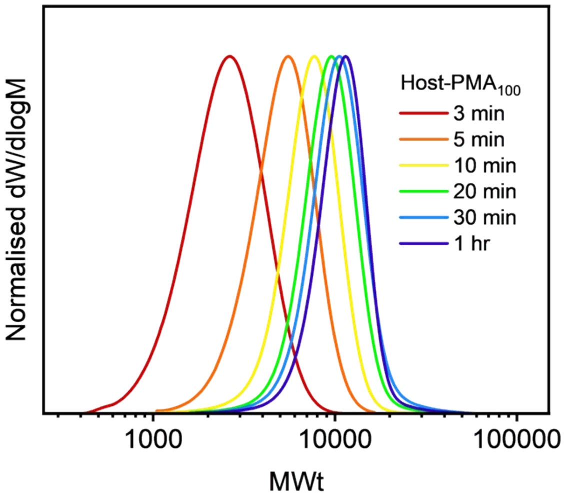

Figure S12: SEC traces showing the evolution of MWt of PMA by photo Cu-RDRP using λ ~ 505 nm.

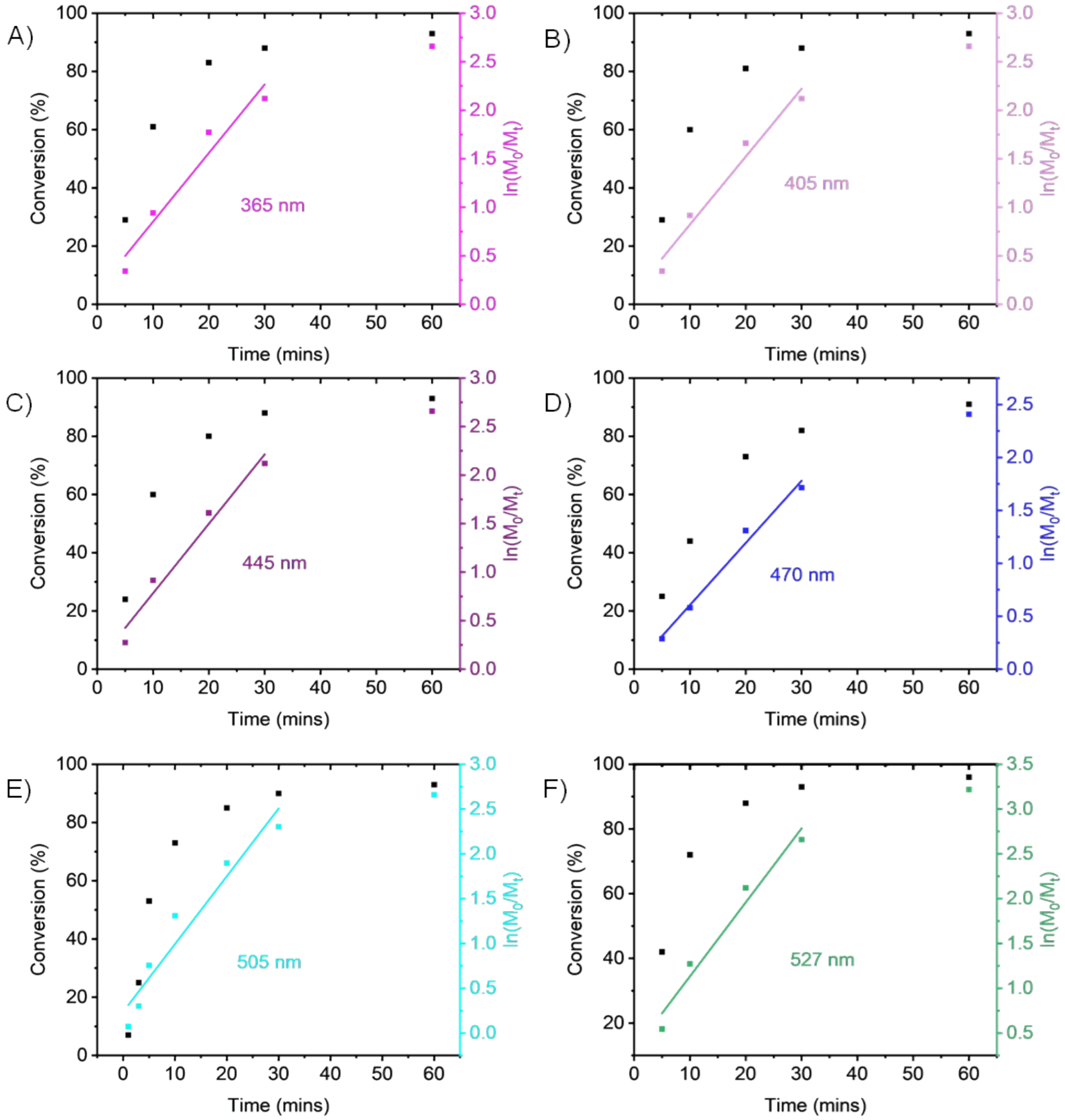

Figure S13: Monomer conversion over time and kinetics of PMA DP_{target} = 100 under irradiation at different wavelengths, A) 365 nm, B) 405 nm, C) 445 nm, D) 470 nm, E) 505 nm and F) 527 nm.

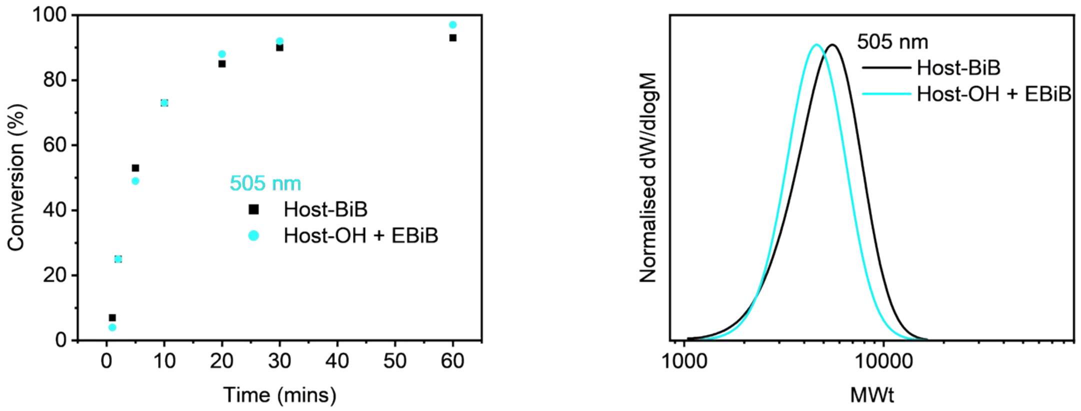

Figure S14: Comparison of PMA DP_{target} = 100, synthesised by photo Cu-RDRP using λ ~ 505 nm with Host-BiB vs EBiB and equivalent amount of Host-OH, with monomer conversion over time (left) and SEC traces after 5 minutes (right).

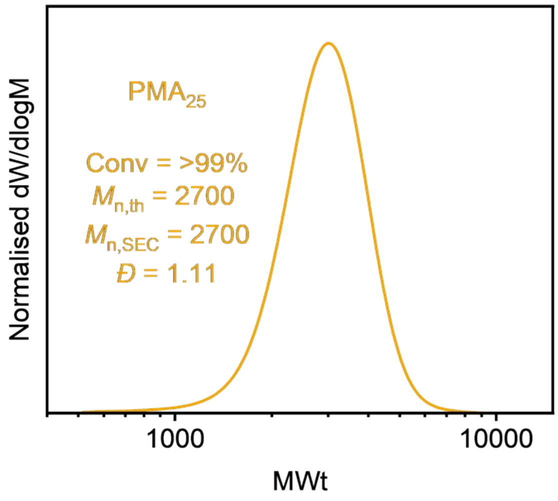

Figure S15: SEC trace of PMA DP_{target} = 25, synthesised by photo Cu-RDRP using λ ~ 505 nm.

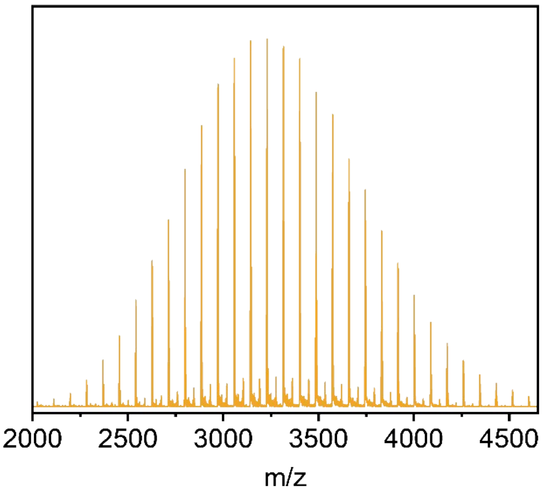

Figure S16: Full MALDI-Tof-MS spectrum of PMA DP_{target} = 25, synthesised by photo Cu-RDRP using λ ~ 505 nm.

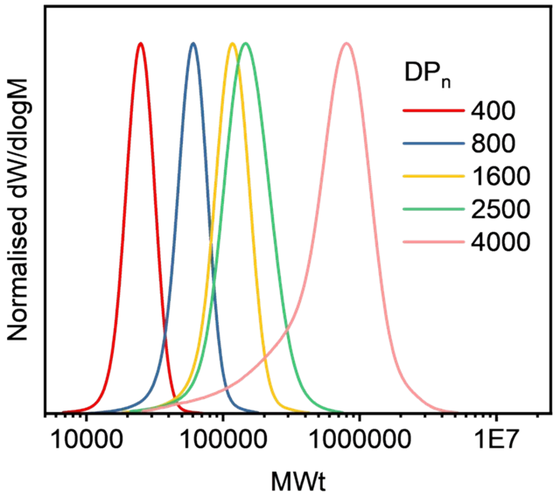

Figure S17: SEC trace of Host-PMA with different DP targets, synthesised by photo Cu-RDRP using λ ~ 505 nm.

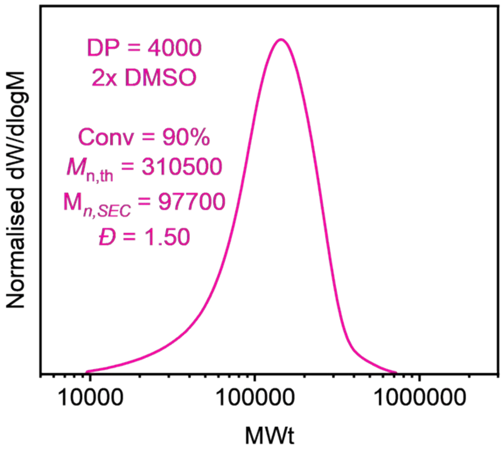

Figure S18: SEC trace of Host-MA$_{4000}$ using 2x the amount of DMSO SEC trace of PMA DP$_{target}$ = 4000, using (monomer: solvent, 1:2, v/v) synthesised by photo Cu-RDRP using λ ~ 505 nm.

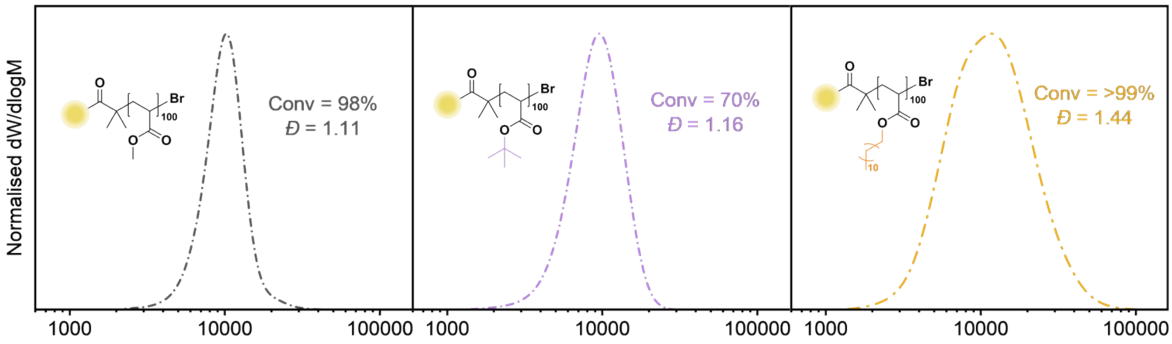

Figure S19: SEC trace of polyacrylates PMA with 50% v/v TFE (left), PtBA with 50% v/v Tol: MeOH 4:1 (middle), and PLA with 50% v/v TFE (right), using Host-BiB, synthesised by photo Cu-RDRP using λ ~ 505 nm.

Table S3: ^{1}H NMR and SEC analysis for all the different Hostasol-BiB initiated polymers.

Polymer[a]	DP	Solvent[b]	Conv (%)[c]	$M_{n,th}$ (g/mol)	$M_{n,SEC}$[d]	Đ
PMA	100	DMSO	>99	9100	10100	1.12
PMA	100	TFE	98	9000	9200	1.11
PEGA	100	DMSO	91	12400	13100	1.13
P(PEGA$_{480}$)	20	DMSO	93	9500	8100	1.13
PtBA	100	TFE	94	12600	13100	1.08
PtBA	100	Tol: MeOH 4:1	70	9500	8100	1.16
PTFEA	100	TFE	92	14700	11600	1.07
PLA	50	TFE	>99	12500	9500	1.44
PLA	50	Tol: MeOH 4:1	80	10100	8000	1.16

[a] [X]: [Host-BiB]: [$CuBr_2$]: [Me_6TREN] = x: 1: 0.02: 0.12 in 50% (v/v) solvent. [b] Reactions with DMSO for 1 hr, with TFE or Tol: MeOH 4:1 for 24 hr. [c] Determined from ^{1}H NMR. [d] Determined from THF SEC analysis.

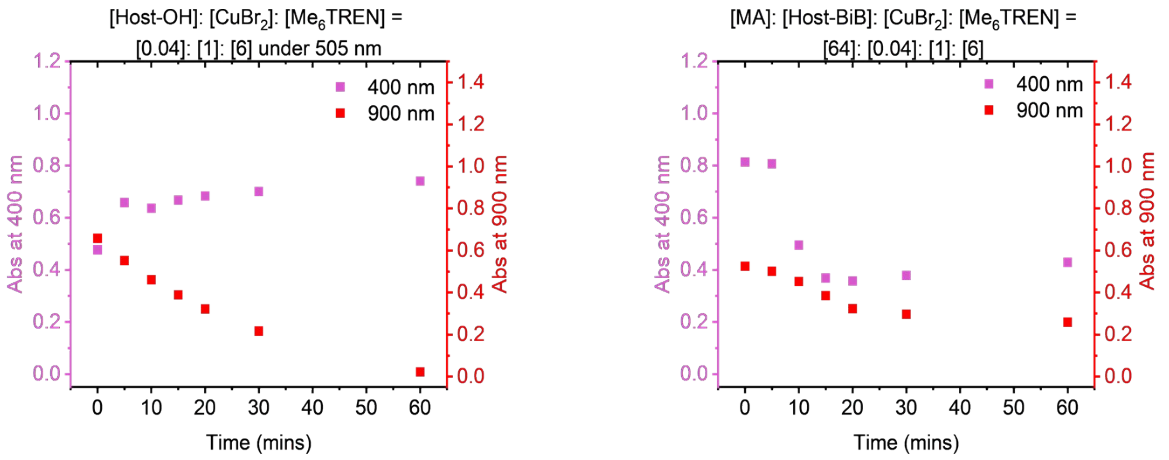

Figure S20: Absorption measurements at 400 nm and 900 nm from the time-dependent UV-Vis spectra of Host-OH and $CuBr_2$/Me_6TREN (left) and MA, Host-BiB and $CuBr_2$/Me_6TREN (right) in DMSO under irradiation at 505 nm.

Table S4: Absorptions and fluorescence integral values for Host-OH and Rhodamine 6G and the calculated quantum yield.

	Entry	Abs at 460 nm	Fluorescence integral	Gradient	Φ_F
Rhodamine 6G	1	0.09462	12624.504	131150.46298	Ref = 0.97
	2	0.07009	9135.7574		
	3	0.05316	7350.72701		
	4	0.03010	4048.36847		
Host-OH	1	0.09292	11990.8491	122788.19209	0.91
	2	0.06539	8784.24655		
	3	0.03941	5554.07937		
	4	0.02068	3116.06188		

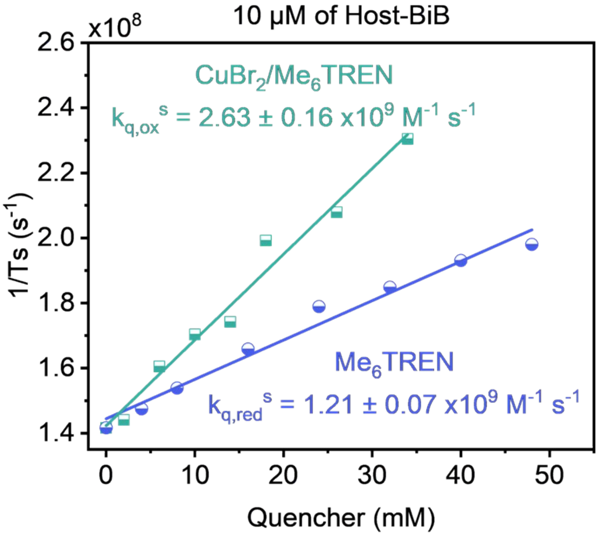

Figure S21: Quenching study plot of the inverse fluorescence lifetime of Host-BiB through TCSPC with λ_{ex} = 462 nm, using $Cu^{II}Br_2$/Me_6TREN (1:1) (green) vs Me_6TREN.

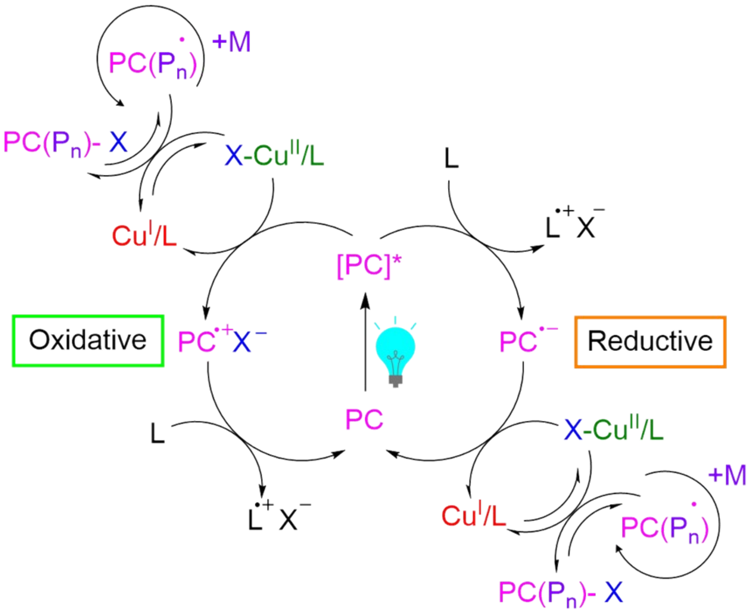

Figure S22: Dual photoredox/copper catalysis proposed mechanism from already published work,[6] now using a dual PC initiator.

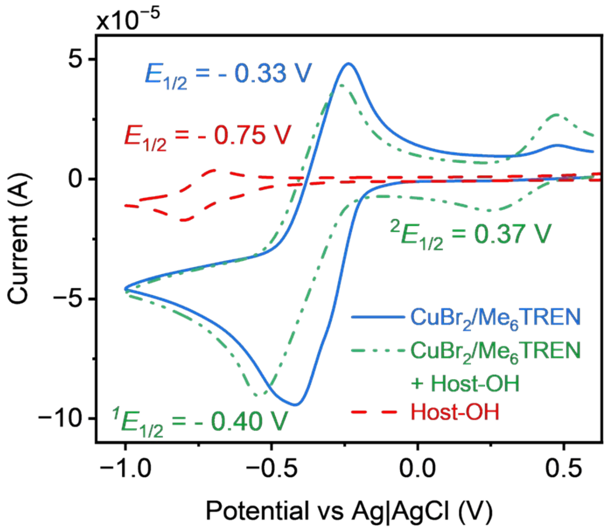

Figure S23: CVs of Host-OH (red), Host-OH and $Cu^{II}Br_2/Me_6TREN$ (green) and $Cu^{II}Br_2/Me_6TREN$ (blue) with 0.001 - 0.01 M concentration, in a 0.1 M tetrabutylammonium hexafluorophosphate solution in DMSO with scan rate 0.1 V/s vs. Ag/AgCl.

References

1 A. J. Limer, A. K. Rullay, V. San, C. Peinado, S. Keely, E. Fitzpatrick, S. D. Carrington, D. Brayden and D. M. Haddleton, *React Funct Polym*, 2006, **66**, 51–64.

2 M. Ciampolini and N. Nardi, *Inorg Chem*, 1966, **5**, 41–44.

3 C. Ma, T. Han, S. Efstathiou, A. Marathianos, H. A. Houck and D. M. Haddleton, *Macromolecules*, 2022, **55**, 9908–9917.

4 Analytical Sales and Services, Inc., https://www.analytical-sales.com/product-category/photoredox-parallel-synthesis/lumidox-ii/, (accessed 4 August 2025).

5 Thorlabs Photodiode Power Sensors (C-Series), https://www.thorlabs.com/newgrouppage9.cfm?objectgroup_id=3328, (accessed 7 August 2025).

6 J. Sobieski, A. Gorczyński, A. Moini Jazani, G. Yilmaz and K. Matyjaszewski, *Angewandte Chemie*, 2025, **64**, e202415785.
